# Phloridine

**Published:** 1843-01

**Authors:** 


*Phloridine.*—This is a new medicine, which is now very highly spoken of by French practitioners as a useful adjunct to our cinchona preparations. It has been used for some years in Germany, Poland, and France. It is extracted from the bark of the roots of the apple-tree and the wild cherry-tree, and is thus prepared: the bark of recent roots is boiled with water sufficient to cover them, for half an hour. This is poured off, and the same quantity is again used; these two fluids are mixed together, and at the end of six hours deposit the phloridine in the form of a deep-red velvety-looking matter.

Lebaudy, the editor of the *Journal des Connaissances Médico-Chirurgicales*, says, "its efficacy is so decided, that we cannot hesitate to class it with the most powerful febrifuges; and it has this advantage over quinine, that it never induces gastralgia."—*Braithwaite's Retrospect, No.* 5.

